# Evolution of Human Brain Size-Associated NOTCH2NL Genes Proceeds toward Reduced Protein Levels

**DOI:** 10.1093/molbev/msaa104

**Published:** 2020-04-24

**Authors:** Gerrald A Lodewijk, Diana P Fernandes, Iraklis Vretzakis, Jeanne E Savage, Frank M J Jacobs

**Affiliations:** m1 Swammerdam Institute for Life Sciences, University of Amsterdam, Amsterdam, The Netherlands; m2 Department of Complex Trait Genetics, Center for Neurogenomics and Cognitive Research, VU University, Amsterdam, The Netherlands; m3 Amsterdam Neuroscience, Complex Trait Genetics

**Keywords:** archaic genomes, brain size, human evolutionary genomics, human-specific genes, segmental duplications, Neanderthal, gene conversion

## Abstract

Ever since the availability of genomes from Neanderthals, Denisovans, and ancient humans, the field of evolutionary genomics has been searching for protein-coding variants that may hold clues to how our species evolved over the last ∼600,000 years. In this study, we identify such variants in the human-specific *NOTCH2NL* gene family, which were recently identified as possible contributors to the evolutionary expansion of the human brain. We find evidence for the existence of unique protein-coding NOTCH2NL variants in Neanderthals and Denisovans which could affect their ability to activate Notch signaling. Furthermore, in the Neanderthal and Denisovan genomes, we find unusual *NOTCH2NL* configurations, not found in any of the modern human genomes analyzed. Finally, genetic analysis of archaic and modern humans reveals ongoing adaptive evolution of modern human *NOTCH2NL* genes, identifying three structural variants acting complementary to drive our genome to produce a lower dosage of NOTCH2NL protein. Because copy-number variations of the *1q21.1* locus, encompassing *NOTCH2NL genes*, are associated with severe neurological disorders, this seemingly contradicting drive toward low levels of NOTCH2NL protein indicates that the optimal dosage of NOTCH2NL may have not yet been settled in the human population.

## Introduction

**Fig. 1.**
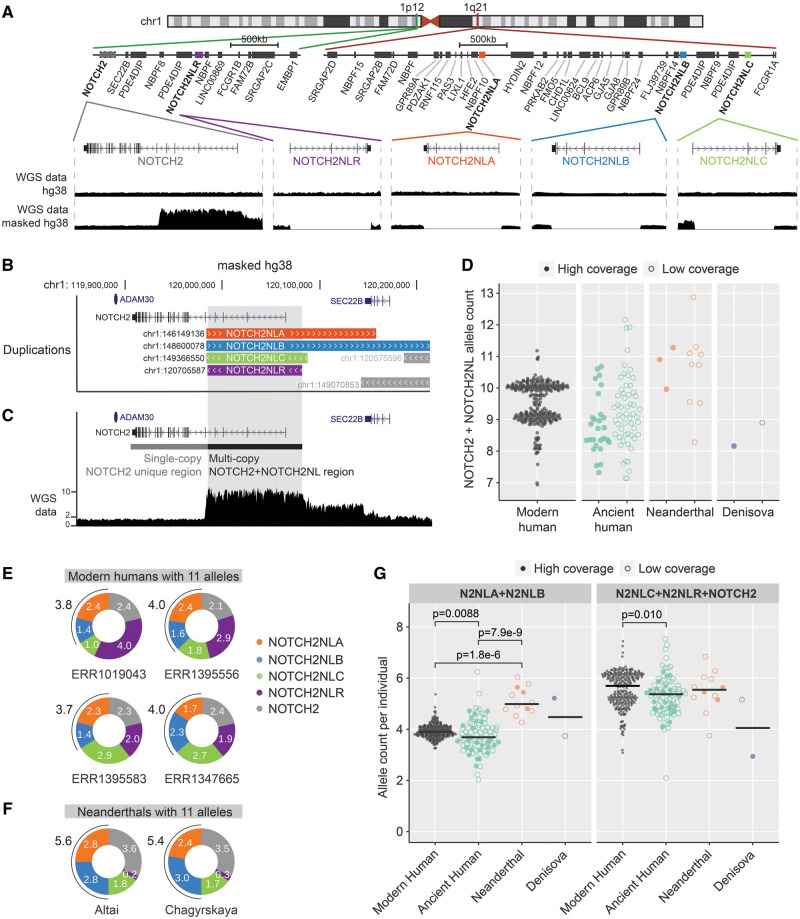
*NOTCH2NL* copy-number analysis in modern human and archaic DNA samples. (*A*) Overview of *NOTCH2* and *NOTCH2NL* loci in the human genome (hg38). Zoom-ins show sequence read depth at the different loci of data mapped on hg38 or masked hg38 reference genome. (*B*) Tracks showing *NOTCH2NL* duplicons from the segmental UCSC browser duplication track in the *NOTCH2* locus. (*C*) Example showing *NOTCH2*- and *NOTCH2NL*-derived sequencing reads piled up on the *NOTCH2* locus on the masked hg38 genome. (*D*) Quantification of *NOTCH2* + *NOTCH2NL* alleles per individual using relative coverage of multicopy/single-copy regions. Modern human, *n* = 279. Ancient human: high (*n* = 27)/low (*n* = 53) coverage; Neanderthal high (*n* = 3)/low (*n* = 9) coverage; Denisova high (*n* = 1)/low (*n* = 1) coverage. (*E*, *F*) *NOTCH2NL* allele counts estimated from the average density of paralog-specific SUNs in modern human outliers (*E*) and Neanderthals (*F*) showing evidence for the presence of 11 alleles in total (two alleles *NOTCH2 *+* *nine alleles *NOTCH2NL*). (*G*) Comparison of allele count grouped by *NOTCH2NLA* + *NOTCH2NLB* (Kruskal–Wallis *P* = 1.8e-8), and *NOTCH2NLR* + *NOTCH2NLC* + *NOTCH2* (Kruskal–Wallis *P* = 0.0055). Kruskal–Wallis test was followed up by Dunn’s test, significant comparisons are indicated in the plots. Modern human, *N* = 279; ancient human, *N* = 80; Neanderthal, *N* = 12; and Denisova, *N* = 2.

The human brain tripled in size after we split from the common ancestor with our closest living relative species, the chimpanzees (Marino 1998; Herculano-Houzel 2009; Hofman 2014). The emergence of human-specific *NOTCH2NL* genes (Fiddes et al. 2018; Florio et al. 2018; Suzuki et al. 2018) coincided with this evolutionary expansion (Holloway et al. 2004; Pollen et al. 2015; Ju et al. 2016; Liu et al. 2017; Johnson et al. 2018; Kalebic et al. 2018) and their association to human brain development put *NOTCH2NL* genes forward as possible contributors to human’s increased brain size. By enhancing Notch signaling, *NOTCH2NL* genes prolong proliferation of neuronal progenitor cells and expand cortical neurogenesis (Fiddes et al. 2018; Florio et al. 2018; Suzuki et al. 2018). *NOTCH2NL* genes are human specific and they emerged after a series of segmental duplications and gene conversion events involving the important neurodevelopmental gene *NOTCH2*. Four *NOTCH2NL* paralogs are present in modern humans: *NOTCH2NLA*, *NOTCH2NLB*, and *NOTCH2NLC* in the *1q21.1* locus (fig. 1*A*) and the pseudogene *NOTCH2NLR* next to the parental *NOTCH2* gene in the *1p12* locus. *NOTCH2NLB* represents the largest duplicon in the cluster, suggesting this was the first *NOTCH2NL* gene present in the genome (fig. 1*B*). Whereas copy-number variation is observed for *NOTCH2NLC* and *NOTCH2NLR* in the healthy human population; the copy number of *NOTCH2NLA* and *NOTCH2NLB* loci is highly stable in modern humans. In fact, *1q21.1* copy-number variations, mediated by breakpoints within the *NOTCH2NLA* and *NOTCH2NLB* genes, are associated with various neurological disorders (Brunetti-Pierri et al. 2008; Mefford et al. 2008; Bernier et al. 2016; Fiddes et al. 2018). These observations suggest that the total number of functional *NOTCH2NLA* and *NOTCH2NLB* alleles may be important for normal neuronal development. Given the highly variable genomic organization of the *1q21.1* locus, important questions remain about the level of variation in *NOTCH2NL* genes in the human population. In addition, it remains elusive whether the number and composition of *NOTCH2NL* genes has changed during recent human evolution. Here, we analyzed the segregation of coding variants in *NOTCH2NL* genes throughout human evolution and compared the composition of each *NOTCH2NL* locus between modern humans and archaic genomes. Our analysis revealed lineage-specific coding variants in each of the genomes of Neanderthals, Denisovans, and modern humans. Intriguingly, we find evidence for ongoing adaptive evolution of multiple structural variants in modern human *NOTCH2NL* genes, acting in synergy and complementary to drive our genome to produce a lower dosage of NOTCH2NL protein. The evolutionary forces mediated by gene conversion Chen et al. 2007, which we find is still ongoing between *NOTCH2NL* loci at a high frequency in modern humans, exemplify how recently duplicated regions in our genome can undergo rapid structural evolution to reach an optimal configuration and functionality. For humans, this may have had important consequences for how a key developmental process such as Notch signaling has evolved in the period after the emergence of *NOTCH2NL* genes and the changes they effectuated on human brain development.

### Additional Copies of *NOTCH2NLA* or *NOTCH2NLB* in Neanderthals

To assess the structural evolution of each of the *NOTCH2NL* loci throughout human evolution, we first assessed the structural variability of *NOTCH2NL* loci in the modern human population. Previous estimations of total *NOTCH2NL* copy number in individuals could not efficiently distinguish between paralogous *NOTCH2NL* loci subject to recent ectopic gene conversion, as observed between *NOTCH2*-*NOTCH2NLR* and between *NOTCH2NLA*-*NOTCH2NLB* (Dougherty et al. 2017; Fiddes et al. 2018). Here, we used an alternative strategy that takes into account gene conversion between paralogous *NOTCH2NL* loci: For each genome, we assessed total number of *NOTCH2NL* alleles based on sequence read coverage and matched this with information about the presence or absence of *NOTCH2NL*-paralog identifying single-unique nucleotides (SUNs) (Sudmant et al. 2010). This provides an accurate assessment of the absolute number of *NOTCH2NL* alleles in each individual genome and a detailed overview of the structural variability of *NOTCH2NL* genes as a consequence of gene conversion (supplementary tables S1–S5, Supplementary Material online). We verified the accuracy of our methodology by showing concordance with previous *NOTCH2NL* assembly–based estimations (supplementary table S6, Supplementary Material online). To assess the total number of *NOTCH2NL* alleles across the human population, the genomes of 279 individuals from the Simons diversity data set (Mallick et al. 2016) were mapped onto a modified hg38 genome in which the *NOTCH2NL* loci are masked (fig. 1*A*). On this modified hg38 genome, all *NOTCH2NL*-derived reads map onto the 5′ side of the *NOTCH2* locus, the part of *NOTCH2* that was originally duplicated forming the *NOTCH2NL* genes (fig. 1*B* and *C*). The coverage analysis reveals that the majority of the human population has ten alleles, encompassing two alleles from *NOTCH2* and two alleles from each of the four *NOTCH2NL* loci (fig. 1*D*). Using the combined sequence coverage and SUN analysis, we determined that each individual contained 4 alleles combined of the highly similar *NOTCH2NLA* and *NOTCH2NLB* genes. The individuals that have nine, eight, or seven alleles were all confirmed as hetero- or homozygotic for *NOTCH2NLC* and *NOTCH2NLR* (supplementary fig. S1*A* and *B*, Supplementary Material online). Four human individuals have one extra allele of *NOTCH2NLC* or *NOTCH2NLR*, indicating that *NOTCH2NL* duplications happen in the healthy human population (fig. 1*E*). Next, we analyzed genomes of ancient humans (0.1k–45k years old) (Keller et al. 2012; Fu et al. 2014, 2016; Gamba et al. 2014; Lazaridis et al. 2014; Olalde et al. 2014; Raghavan et al. 2014; Rasmussen et al. 2014, 2015; Seguin-Orlando et al. 2014; Skoglund et al. 2014, 2017; Günther et al. 2015, 2018; Jones et al. 2015, 2017; Cassidy et al. 2016; Martiniano et al. 2016; Schiffels et al. 2016; Saag et al. 2017; Bhattacharya et al. 2018; de la Fuente et al. 2018; Krzewińska et al. 2018; Valdiosera et al. 2018; Wright et al. 2018; Sánchez-Quinto et al. 2019), Neanderthals (38k–100k years old) (Green et al. 2010; Prüfer et al. 2014, 2017; Hajdinjak et al. 2018; Slon et al. 2018; Mafessoni et al. 2020), and Denisovans (64k–100k years old) (Meyer et al. 2012; Slon et al. 2017). Although most of the ancient human genomes display *NOTCH2NL* allele numbers that fall within the range of modern humans, several of the 12 available Neanderthal genomes show increased coverage, which indicates they contained an extra *NOTCH2NL* duplication (fig. 1*D*). Whereas the combined copy number of *NOTCH2NLA* and *NOTCH2NLB* is highly stable in healthy modern humans, SUN-based copy-number estimation suggests that Neanderthals carried an extra duplication of the *NOTCH2NLA* or *NOTCH2NLB* gene (fig. 1*F* and *G* and supplementary fig. S1*C*, Supplementary Material online). Whether this is a gain in Neanderthal or a loss in modern humans remains elusive. In addition, all Neanderthal genomes showed evidence of extensive gene conversion between *NOTCH2* and *NOTCH2NLR* (supplementary fig. S1*C*, Supplementary Material online), a phenomenon observed only occasionally in modern humans (supplementary fig. S1*D* and *E*, Supplementary Material online).

### Neanderthals and Denisovans Carried Specific NOTCH2NL Variants

**Fig. 2.**
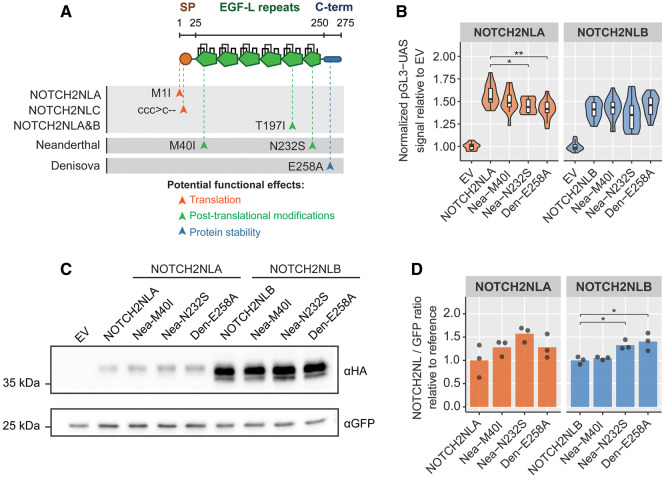
Characterization of archaic *NOTCH2NL* coding variants. (*A*) Overview of modern human, Neanderthal-specific, and Denisovan-specific coding variants. (*B*) Coculture NOTCH2 reporter assay testing Neanderthal and Denisovan variants reconstructed in the human *NOTCH2NLA* cDNA (*n* = 15 in three experiments, analysis of variance (ANOVA) *P* = 0.002, followed by Tukey’s test), or the human *NOTCH2NLB* cDNA (*n* = 20 in four experiments, ANOVA *P* = 0.07). (*C*) Western blot analysis of Neanderthal and Denisovan variants. Plasmids were transfected in equimolar amounts. (*D*) Quantification of protein level from three independent experiments for NOTCH2NLA (ANOVA *P* = 0.12) and NOTCH2NLB (ANOVA *P* = 0.006, followed by Tukey’s test). Asterisks indicate significant values from Tukey’s post hoc tests: *P < 0.05 and **P < 0.01.

We next investigated whether the archaic genomes contained any coding sequence variants that may have encoded unique NOTCH2NL protein variants. Despite an overall high similarity (99.9%) between human and Neanderthal/Denisovan *NOTCH2NL* exons, we found evidence for two Neanderthal-specific coding variants and one Denisova-specific coding variant (fig. 2*A*). In the Altai Neanderthal genome, an ATG > ATA (M40I) missense variant (NOTCH2NL^Nea-M40I^) is detected in 17/242 (∼8%) of the sequencing reads corresponding to one allele out of the nine *NOTCH2NL* alleles found in Altai Neanderthals. The second Neanderthal-specific variant is a N232S missense variant (NOTCH2NL^Nea-N232S^) detected in 28/177 (∼18%) of sequencing reads, corresponding to two alleles. This variant is also present in the genomes of the Vindija and Chagyrskaya Neanderthals and most of the low-coverage Neanderthal genomes, indicating the NOTCH2NL^Nea-N232S^ variant was a common variant in the Neanderthal lineage. In the Denisova3 genome, a Denisovan-specific E258A missense variant (NOTCH2NL^Den-E258A^) is found in 38/203 (∼19%) of the sequencing reads, also corresponding to two alleles. Importantly, none of these variants are found in the 279 modern human genomes of the Simons diversity data set. Interestingly, the NOTCH2NL^Nea-N232S^ was found as a rare variant in modern humans (rs375605753) with an allele frequency of 0.0002 in UK Biobank exome sequencing data (*N* = 49,593), suggesting this was one of the Neanderthal-derived genetic variants that was contributed to the human genome after interbreeding with Neanderthals (Dannemann and Racimo 2018). It should be noted that the highly fragmented assemblies of archaic genomes prevents us from making solid claims about which *NOTCH2NL* paralog each of these archaic variants reside in. Taking this into account, we assessed the potential functional implications of the Neanderthal and Denisova variants by reconstructing the archaic NOTCH2NL variants in NOTCH2NLA and NOTCH2NLB for functional testing in a previously established Notch signaling reporter assay (Groot et al. 2014; Habets et al. 2015; Fiddes et al. 2018) (supplementary fig. S2*A*, Supplementary Material online). Surprisingly, the introduction of the Nea-N232S and Den-E258A into human NOTCH2NLA showed a modest but significant decrease in potency to enhance Notch signaling (fig. 2*B*). To find an explanation for the functional divergence of the archaic NOTCH2NL variants, we investigated the potential structural implications in more detail (supplementary fig. S2*B*, Supplementary Material online). The Neanderthal M40I variant is located in EGF-L domain 1 and disrupts the predicted start codon of NOTCH2NLA. The Neanderthal N232S variant is located in EGF-L domain 6, which is fully conserved between NOTCH paralogs and between species (supplementary fig. S2*C*, Supplementary Material online). The N232 residue is part of an important motif for glycosylation, a posttranslational modification which mediates EGF-L folding (Takeuchi et al. 2017) and NOTCH–ligand interactions (Jafar-Nejad et al. 2010) (supplementary fig. S2*D*, Supplementary Material online). As such, the N232S variant is predicted to alter NOTCH2NL protein interaction dynamics or protein stability (supplementary fig. S2*E*, Supplementary Material online). Indeed, the corresponding rare single-nucleotide polymorphism (SNP) in modern humans (rs375605753) is predicted to be deleterious (Pejaver et al. 2017). The Denisova E258A variant is located in the C-terminal domain of NOTCH2NL, an intrinsically disordered region known to play a role in protein stability (Duan et al. 2003; Fiddes et al. 2018). Analysis using IUPred2A (Mészáros et al. 2018) suggests that this substitution alters the state of the NOTCH2NL C-terminal domain, potentially affecting protein stability (supplementary fig. S2*E* and *F*, Supplementary Material online). In support of this, a modest increase in protein level was observed for the Den-E258A and Nea-N232S variants introduced into human NOTCH2NLB (fig. 2*C* and *D*). This suggests that these archaic variants positively affected protein translation or stability. Altogether, Denisovans and Neanderthals carried alleles in their genome which are likely to have affected the function of their *NOTCH2NL* genes.

### Variants in Exon1 of *NOTCH2NL* Genes Determine NOTCH2NL Protein Levels

**Fig. 3.**
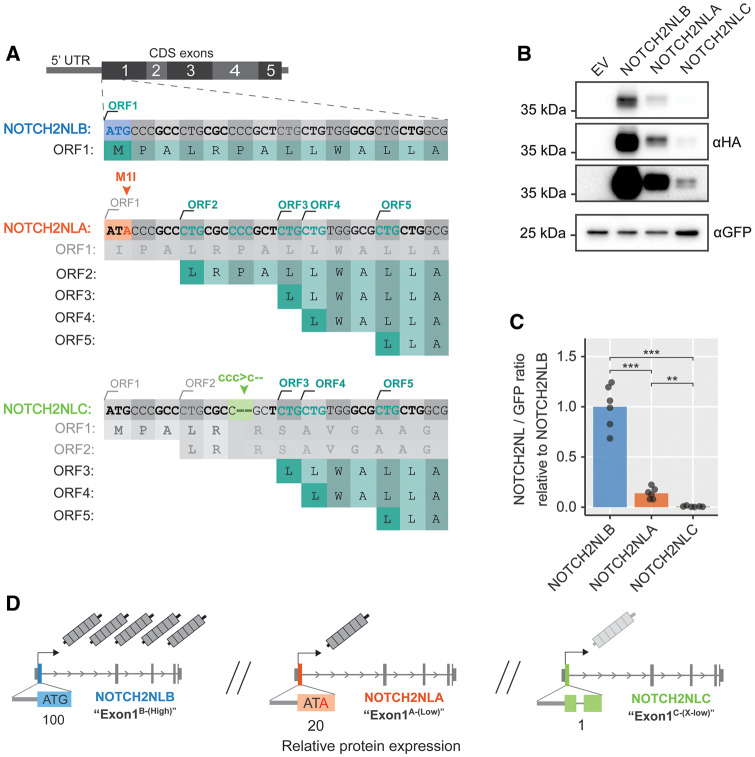
*NOTCH2NL* Exon1 variants define protein expression level. (*A*) Overview of *NOTCH2NL* Exon1 variants in *NOTCH2NLB* (blue), *NOTCH2NLA* (orange), and *NOTCH2NLC* (green). The ORFs produced by each variant are indicated in dark green. (*B*) Western blot analysis of *NOTCH2NL* Exon1 coding variants. (*C*) Quantification of protein expression level from equimolar quantities of *NOTCH2NLB*, *NOTCH2NLA*, or *NOTCH2NLC* full-length cDNAs. Data from six independent experiments, analysis of variance (ANOVA) (Welch corrected) *P* = 2.7e-05, followed Games–Howell test: **P < 0.01 and ***P < 0.001. (*D*) Overview of NOTCH2NL loci, the configuration of the Exon1 variants, and the relative levels of NOTCH2NL protein they produce.

Unexpectedly, we noticed that the NOTCH2NLA^Nea-M40I^ variant, predicted to lack the first 83 amino acids, was not different in size from NOTCH2NLB. Likewise, no decrease in protein size was observed for NOTCH2NLA, predicted to lack the first 39 amino acids. Analysis of multiple 5′ truncated *NOTCH2NL* cDNAs reveals that instead of the conventional ATG initiation sites on positions M40 and M84, multiple unconventional CTG start sites in the 5′ side of *NOTCH2NL* drive translation of NOTCH2NLA and NOTCH2NLA^Nea-M40I^ proteins (Kearse and Wilusz 2017) (fig. 3*A* and supplementary fig. S3*A*–*G*, Supplementary Material online). As a result and as opposed to what is predicted by gene models, human *NOTCH2NLA* and Neanderthal *NOTCH2NLA^Nea-M40I^* encode almost full-length NOTCH2NL proteins with a functionally intact N-terminal signal peptide. Importantly, our analysis also reveals that the usage of unconventional translation initiation sites has major consequences for the level of NOTCH2NL protein produced by each of the *NOTCH2NL* genes. *NOTCH2NLA*, which lacks the first start codon produces a 5-fold lower level of NOTCH2NL protein compared with NOTCH2NLB (fig. 3*A*–*C*). *NOTCH2NLC* is also forced to use downstream CTG sites for translation initiation and gives rise to normal-sized NOTCH2NL protein (fig. 3*B*). However, due to the combination of the *NOTCH2NLC*-characteristic 2-bp deletion and upstream open-reading frames (ORFs), the expression level of NOTCH2NLC is extremely low, at only 1% compared with NOTCH2NLB (fig. 3*C*). These new insights reveal that the level of NOTCH2NL protein generated by each of the genes is predominantly dependent on the presence or absence of three specific coding variants in Exon1 (fig. 3*D*). Compared with the *NOTCH2NLB* configuration of Exon1 (Exon1^B-(High)^-variant) which produces high levels of NOTCH2NL protein, the M1I substitution in NOTCH2NLA (Exon1^A-(Low)^-variant) produces 5-fold less NOTCH2NL protein. The configuration of *NOTCH2NLC*, which has the 2-bp deletion in Exon1, (Exon1^C-(X-low)^-variant) results in extremely low levels of NOTCH2NL protein. Importantly, ectopic gene conversion between *NOTCH2NL* loci can result in transfer of Exon1-variants from one *NOTCH2NL* gene to another. As a consequence, the total dosage of NOTCH2NL protein in each individual may not be defined by the copy number of each of the *NOTCH2NL* genes, but by the level of Exon1-variant carry-over via gene conversion between *NOTCH2NL* genes.

### Unusual Configuration of *NOTCH2NL* Genes in the Denisova3 Genome

**Fig. 4.**
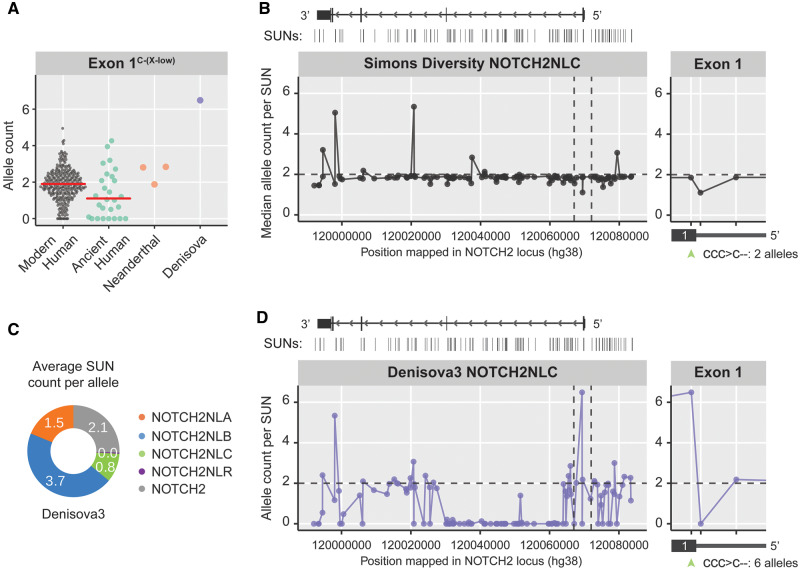
*NOTCH2NLC* configuration in Denisova3 compared with modern humans. (*A*) Plot showing the Exon1^C-(X-low)^ allele count for modern humans, ancient humans, Neanderthals, and Denisovan. Note the unusual allele count for Denisovan. (*B*) Modern human’s median allele count plotted for each of the *NOTCH2NLC*-specific SUNs distributed along the *NOTCH2NL* locus. Vertical dashed lines indicate the region around Exon1. Zoom-in shows SUN count in Exon1, including the Exon1^C-(X-low)^ variant indicated by green arrowhead. (*C*) *NOTCH2NL* allele counts in the Denisova3 genome, estimated from the average density of paralog-specific SUNs. (*D*) Denisova3 allele count plotted for each of the *NOTCH2NLC*-specific SUNs distributed along the *NOTCH2NL* locus. Zoom-in shows *NOTCH2NLC* SUN count in Exon1, including the Exon1^C-(X-low)^ variant as indicated by green arrowhead.

To assess the extent to which gene conversion influences the distribution of Exon1-variants between *NOTCH2NL* genes, we investigated the distribution of SUNs across the *NOTCH2NL* loci. First, we analyzed modern human *NOTCH2NLC* for evidence of gene conversion. Analysis of the Exon1 configuration of *NOTCH2NL* genes reveals that most modern humans contain two *NOTCH2NLC*-derived Exon1^C-(X-low)^-variants (fig. 4*A*), present in both alleles of *NOTCH2NLC*. Furthermore, an equal distribution was found for *NOTCH2NLC* SUNs across the *NOTCH2NL* locus in most modern human individuals (fig. 4*B*), suggesting that gene conversion between *NOTCH2NLC* and other *NOTCH2NL* loci does not commonly happen. A similar pattern was found in Neanderthals and ancient humans (fig. 4*A* and supplementary fig. S4*A* and *B*, Supplementary Material online). This indicates that the majority of Neanderthal, archaic human, and modern human genomes have two *NOTCH2NLC* alleles carrying the Exon1^C-(X-low)^-variant. The Denisova3 genome however, shows a strikingly different pattern.

The presence of *NOTCH2NL*-paralog-specific SUNs across the *NOTCH2NL* loci shows that *NOTCH2NLA*, *NOTCH2NLB*, and *NOTCH2NLC* genes are present in the Denisova3 genome (fig. 4*C*). Based on the complete absence of *NOTCH2NLR* SUNs and a total coverage representative of only six *NOTCH2NL* alleles (fig. 1*D*), it is likely the Denisova3 genome had a homozygous deletion of *NOTCH2NLR*. Remarkably, despite good coverage of the Exon1 region in the Denisova3 genome (36X), all *NOTCH2NL*-derived reads from Exon1 carry the *NOTCH2NLC*-derived Exon1^C-(X-low)^-variant (fig. 4*D*). This implies that all six Denisovan *NOTCH2NL* alleles produced NOTCH2NL protein at an extremely low level. Unfortunately, the lack of other high-coverage Denisovan genomes prevents us from assessing whether this is an individual-specific genotype or whether similar *NOTCH2NLC* gene conversions were frequent in the Denisovan population. Importantly, this pattern of Exon1^C-(X-low)^-variant distribution in Denisovan *NOTCH2NL* genes, or anything similar to it, has not been observed in any of the analyzed genomes of Neanderthals or healthy modern humans (supplementary fig. S4*C*, Supplementary Material online).

### Evolution of Modern Human *NOTCH2NL* Genes Trends toward Lower NOTCH2NL Levels

**Fig. 5.**
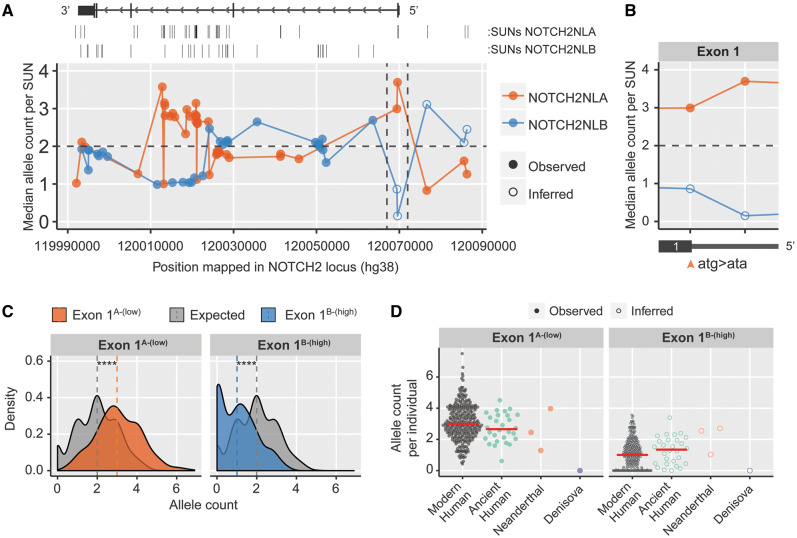
Exon1 variant frequencies in modern human and ancient genomes. (*A*) Median allele count for each of the *NOTCH2NLA-* and *NOTCH2NLB*-specific SUNs along the *NOTCH2NL* locus in Simons diversity genomes (*N* = 279). (*B*) Zoomed in region of Exon1, orange arrowhead indicates Exon1^B-(High)^ (ATG)/Exon1^A-(Low)^ (ATA) variant positions. (*C*) Distribution of Exon1^A-(Low)^ and Exon1^B-(High)^ (inferred) variants in Simons diversity genomes. Expected distribution models equal frequency of both variants. Vertical dashed lines indicate medians. *N* = 279, Kolmogorov–Smirnov test: *P* < 2e-16. (*D*) Analysis of Exon1^A-(Low)^ and Exon1^B-(High)^ (inferred) variant frequency in modern humans and archaic genomes. Red lines indicate medians.

Even though *NOTCH2NLA* and *NOTCH2NLB* are capable of producing a structurally similar NOTCH2NL protein, the protein levels they produce differ by 5-fold. In the SUN analysis, we find evidence of extensive gene conversion between the *NOTCH2NLA* and *NOTCH2NLB* loci: The median SUN depth shifts in favor of either allele in different regions of the loci, indicating that parts of the *NOTCH2NLA*-sequence are frequently overwritten by *NOTCH2NLB*-sequence and vice versa (fig. 5*A*). Most regions with a strong shift in distribution of *NOTCH2NLA* or *NOTCH2NLB* SUNs are intronic, not predicted to impact the structure and level of NOTCH2NL protein. However, the configuration of Exon1 in *NOTCH2NLA* and *NOTCH2NLB* shows a median allele depth strongly in favor of the Exon1^A-(Low)^-variant (fig. 5*B*). This is striking because it suggests that the vast majority of the population carries three or four alleles with the *NOTCH2NLA*-derived Exon1^A-(Low)^-variant and only one or zero alleles with the *NOTCH2NLB*-derived Exon1^B-(High)^-variant (fig. 5*C*). The shift in Exon1^A-(Low)^-variant distribution was confirmed in 49,593 exomes from the UK Biobank (Van Hout et al. 2019) (supplementary fig. S5*A*, Supplementary Material online) and was also observed in the genomes of ancient modern humans (fig. 5*D*). The observed imbalance in distribution of Exon1-variants indicates that the Exon1^B-(High)^-variant, producing the highest levels of NOTCH2NL protein, is being lost or actively being purged out from the modern human population by gene conversion. The increase of the Exon1A-(Low) variant frequency to three or four alleles per individual is likely caused by gene conversion between the *NOTCH2NLA* and *NOTCH2NLB* loci, which can occur during meiosis or in early embryonic development for very unstable loci (Chen et al. 2007; Bruder et al. 2008; Vadgama et al. 2019).

### Spreading of Modern Human-Specific Deleterious Variants Indicates Strong Compensatory Mechanisms

**Fig.6.**
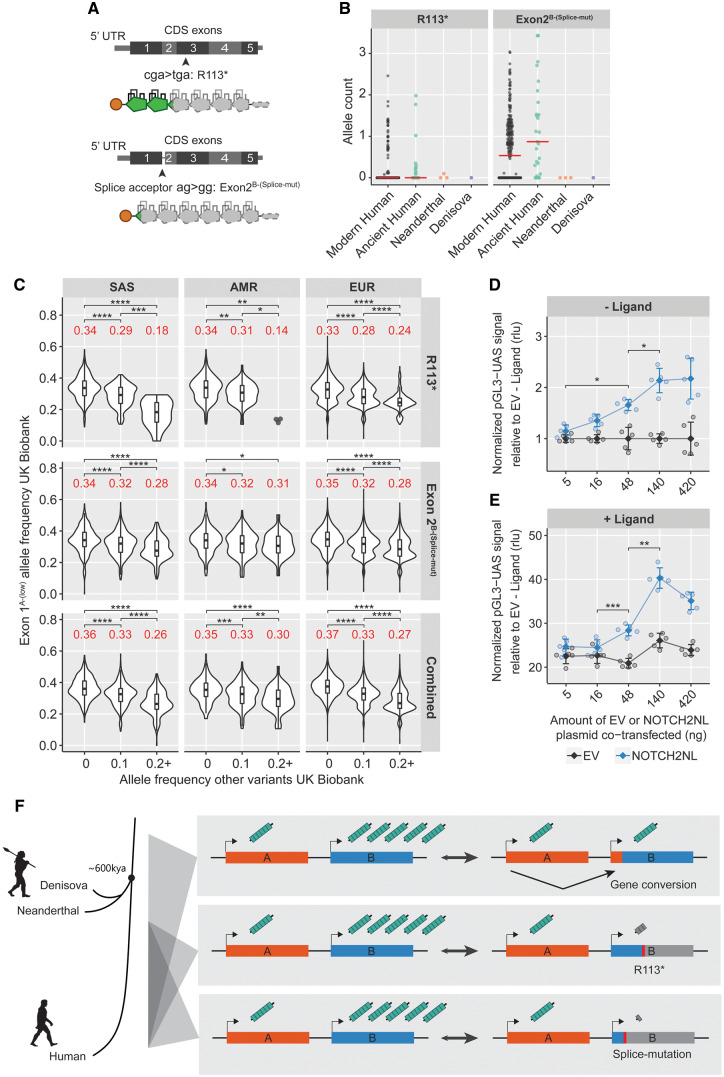
Additional deleterious *NOTCH2NL* variants are present specifically in humans. (*A*) Overview of the R113* and Exon^2B-(Splice-mut)^ deleterious variants on NOTCH2NL protein structure. (*B*) R113* and Exon^2B-(Splice-mut)^ allele count in modern human and archaic genomes. (*C*) UK Biobank data for SAS, AMR, and EUR ancestries showing association of Exon1^A-(Low)^ frequency with R113* frequency, Exon2^B-(Splice-mut)^ frequency, and their combined total grouped by ancestry. R113* Kruskal–Wallis: SAS *P* = 2.2e-16, AMR *P* = 7.8e-5, and EUR *P* = 2.2e-16. Exon2^B-(Splice-mut)^ Kruskal–Wallis: SAS *P* = 4.6e-15, AMR *P* = 0.04, and EUR *P* = 1.1e-15. Combined Kruskal–Wallis: SAS *P* = 2.2e-16, AMR *P* = 9.9e-7, and EUR *P* = 2.2e-16. Significant groups were followed by Dunn’s test. (*D*, *E*) Dose–response curve using increasing amounts of NOTCH2NL in the coculture NOTCH2 reporter assay. (*D*) NOTCH2 expressing cells are cocultured with U2OS (− ligand, analysis of variance [ANOVA] *P* = 7.4e-7, followed by Tukey’s test: *P < 0.05) or (*E*) U2OS-JAG2 (+ ligand, ANOVA *P* = 4.7e-9, followed by Tukey’s test: **P < 0.01 and ***P < 0.001) cells. *n* = 5 per condition, displayed as mean ± SD. (*F*) General overview schematic showing the impact of variants in NOTCH2NL genes on the production of NOTCH2NL protein and the time/lineage where they were segregating. Asterisks indicate significant values from Dunn’s post hoc tests: *P < 0.05, **P < 0.01, ***P < 0.001, and ****P < 0.0001. EAS *N* = 266, SAS *N* = 1,174, AMR *N* = 444, EUR *N* = 46,578, and AFR *N* = 1,087.

Despite the relatively high abundance of Exon1^A-(Low)^ variants in *NOTCH2NLA* and *NOTCH2NLB*, some individuals still carry a relatively high number of Exon1^B-(High)^ variants. We found that individuals with a relatively high number of the Exon1^B-(High)^ variant and low number of the Exon1^A-(Low)^ variant often carry a nonsense SNP (R113*) in NOTCH2NLB, which leads to a premature stop-codon and a severely truncated NOTCH2NL protein (fig. 6*A*). In addition, we found another variant in the splice acceptor sequence of exon 2 (Exon^2B-(Splice-mut)^) (fig. 6*A* and supplementary fig. S6*A*, Supplementary Material online). This variant falls outside the coding region and therefore was not detected before. The AG > GG mutation is predicted to lead to an alternative splicing event, resulting in a frameshift and truncation of NOTCH2NL proteins at amino acid 30 (Dougherty et al. 2018). On hg38, this variant is annotated in *NOTCH2NLB* and it is present at a high allelic frequency in human genomes from the Simons diversity data (supplementary fig. S6*B*, Supplementary Material online) and the UK Biobank (supplementary fig. S6*C*, Supplementary Material online). The R113* variant is less frequently observed. Surprisingly, the splice acceptor variant Exon^2B-(Splice-mut)^ and the R113* mutation were not found in any of the currently available Neanderthal or Denisovan genomes (fig. 6*B* and supplementary fig. S6*B*, Supplementary Material online) and are therefore recently evolved human lineage-specific adaptations. Both loss-of-function variants appear to be common in the South-Asia (SAS), American (AMR), and European (EUR) ancestries and are only sporadically present in East-Asian (EAS) or African (AFR) ancestries in the UK Biobank data (fig. 6*B* and supplementary fig S6*C* and *D*, Supplementary Material online). Segregation of the disruptive alleles appeared to be nonrandom because we found a clear correlation between the individual’s number of Exon1 ^A-(low)^ or Exon1^B-(High)^ variants and the presence of disruptive R113* and Exon^2B-(Splice-mut)^ mutations: Individuals with a relatively high number of the Exon1^B-(High)^ variant, often carry one or two alleles of the disruptive R113* mutation in *NOTCH2NLB* (fig. 6*C*-upper panel and supplementary fig. S6*C* and *D*, Supplementary Material online). A strikingly similar pattern was observed for the Exon^2B-(Splice-mut)^ mutation (fig. 6*C*-middle panel and supplementary fig. S6*C* and *D*, Supplementary Material online). Conversely, individuals with a relatively higher number of Exon1^A-(Low)^ variants are more likely to lack either the R113* or splice acceptor mutations in *NOTCH2NLB* (fig. 6*C*-lower panel and supplementary fig. S6*C* and *D*, Supplementary Material online). In the EAS population, the more sporadic occurrence of both disruptive NOTCH2NL variants correlates with an overall higher Exon1^A-(Low)^ frequency instead (supplementary fig. S6*D* and *E*, Supplementary Material online). This reveals a complex pattern of *NOTCH2NL* configurations, where multiple structural variants in *NOTCH2NLB*, the gene that has the largest contribution to the overall NOTCH2NL levels, seem to act complementary to reduce NOTCH2NL protein levels. In the Simons diversity data set, we observe highly similar patterns, but this analysis lacked statistical power due to the relatively small sample size per ancestry group (supplementary fig. S7*A*–*C*, Supplementary Material online). Taken together, our findings suggest that a relatively high load of the Exon1^B-(High)^ variant often co-occurs with the presence of nonsense variants in *NOTCH2NLB.* Our data suggest that on the individual’s genome level, gene conversion of the Exon1^B-(High)^ variant into the Exon1^A-(Low)^ variant acts in concert with nonsense variants in *NOTCH2NLB* to reduce overall NOTCH2NL protein level. This seems particularly relevant because we observe a strong dosage-dependent effect of NOTCH2NL on Notch signaling activation (fig. 6*D* and *E*), indicating that NOTCH2NL dosage is tightly associated with its functional output, which in the brain is controlling cortical neurogenesis. Altogether, the identification of Neanderthal-, Denisovan-, and modern human-specific coding variants and their complementary functional impact on NOTCH2NL protein levels suggests that the optimal level of NOTCH2NL protein has been under strong selective pressure in recent human evolution and is still being optimized in the human population (fig. 6*F*).

## Discussion

The detection of multiple lineage-specific coding variants and the rapid spread of some of them throughout modern human genomes shows that the structure of human *NOTCH2NL* genes has been subject to ongoing adaptive evolution since the split of modern humans, Neanderthals, and Denisovans from our common ancestor ∼600,000 years ago. This is corroborated by the presence of additional copies of *NOTCH2NLA* or *NOTCH2NLB* in Neanderthal genomes and the unusual configuration of six *NOTCH2NLC*-derived Exon1^C-(X-low)^ variants in the Denisova3 genome. Notably, none of the 279 modern human individuals analyzed in detail in this study showed similar configurations and it is questionable whether such configurations are found in the healthy human population. This raises questions about the health state of the juvenile Denisovan female from the Denisova3 genome, but because the DNA was isolated from a finger bone, information about her physical condition or cause of death is lacking (Meyer et al. 2012). Although our data indicate no major role for *NOTCH2NLC* in normal development due to its low protein expression levels and common loss of one allele, recent studies describe repeat expansions in the 5′UTR *NOTCH2NLC* genes linked to neurodegenerative disorders (Deng et al. 2019; Ishiura et al. 2019; Okubo et al. 2019; Sone et al. 2019; Hayashi et al. 2020; Jiao et al. 2020; Sun et al. 2020). So, it is possible that this repeat expansion leads to disease via a gain-of-function mechanism. For example, it could be that the repeat expansions in *NOTCH2NLC* lead to an N-terminally extended ORF, which in turn may cause aberrantly high expression of NOTCH2NL or production of toxic NOTCH2NL protein variants. Further experiments regarding these possibilities are necessary to understand the mechanisms that underlie the reported disease phenotypes.

Our data suggest that gene conversion still plays a central role in exchanging coding variants between *NOTCH2NLA* and *NOTCH2NLB*. Strikingly, we found that the majority of the population carries three or four *NOTCH2NLA*-derived Exon1^A-(Low)^-variants, which is associated with a substantial reduction in NOTCH2NL protein level. The fact that about 40% of individuals lack the *NOTCH2NLB*-derived Exon1^B-(High)^-variant completely could indicate that the high level of NOTCH2NL protein producing variant is slowly being purged from the human genome. We found that this is not the only evolutionary force at play: Next to the Exon1 variants, there are two other deleterious variants, R113* and Exon^2B-(Splice-mut)^, that reduce the dosage of functional NOTCH2NL protein. Remarkably, these deleterious variants are more often found in individuals with higher Exon1^B-(High)^ frequency, indicating that they provide complementary genetic strategies to decrease NOTCH2NL dosage. The R113* and Exon^2B-(Splice-mut)^ variants are exclusively present in modern humans and are therefore human-specific adaptations that result in reduced NOTCH2NL protein levels. The driving force behind the evolutionary trend to lower levels of NOTCH2NL protein remains elusive. Phylogenetic comparisons or d*N*/d*S* analysis are traditionally used to assess if such variation is significantly associated with evolutionary selection. Because of the absence of functional nonhuman orthologs required to do these comparisons, it is not possible to apply these approaches for analysis of *NOTCH2NL* genes. In addition, frequent and ongoing gene conversion between *NOTCH2* and *NOTCH2NL*-containing loci also hampers this analysis when trying to make comparisons with the truncated *NOTCH2NL* pseudogenes in chimpanzee and gorilla. The high frequency of multiple variants that decrease the available levels of NOTCH2NL protein suggests that *NOTCH2NL* genes have been under selection to counteract high levels of NOTCH2NL expression. Whereas a high frequency of loss-of-function alleles in a population could in principle argue against an essential function of the gene in question and could progress to a complete loss of functional alleles in the future, our data indicate that this is not the case for *NOTCH2NL* genes: Based on the high frequency of loss-of-function variants in *NOTCH2NL* genes in modern humans, it would be expected that a decent proportion of the population would have a genomic configuration without any functional *NOTCH2NL* allele. This is clearly not the case, as the skewed allele distributions that we report points toward purifying selection in order to maintain at least one functional copy of *NOTCH2NL*. This suggests that in present day humans, a certain minimal level of NOTCH2NL protein is required for normal human development. The observed evolutionary changes in *NOTCH2NL* composition could be the result of evolutionary adaptations that took place in any of the tissues where *NOTCH2NL* is expressed, including the developing brain. Even though this remains speculative at the moment, the trend toward lower levels of NOTCH2NL proteins in the human lineage could be correlated to previous observations suggesting a progressive reduction of human brain size that started about 60,000 years ago (Henneberg 1988; Bednarik 2014).

Effectively, NOTCH2NL dosage, which is the total of protein produced by all *NOTCH2NL* loci, may vary between individuals but seems to stay within certain upper and lower ranges. Our new insights regarding the effect of Exon1 variants on NOTCH2NL protein levels may also help in understanding to what extent *NOTCH2NL* genes contribute to *1q21.1* Copy-number variation (CNV)-related phenotypes. Specifically for NOTCH2NL-mediated effects, like potentiating NOTCH signaling, CNVs of an allele carrying the Exon1^B-(High)^ variant may have a much larger effect than CNVs of an allele carrying the Exon1^A-(Low)^ variant. Identifying which *NOTCH2NL* loci are affected by gain and loss of alleles will have to be complemented by distribution analysis of Exon1^A-(Low)^, Exon1^C-(X-low)^, R113*, and Exon^2B-(Splice-mut)^ variants as they are major determinants of NOTCH2NL levels. The realization that gene conversion between functionally different *NOTCH2NL* genes can contribute to the rapid adaptation of the human species to establish lower levels of NOTCH2NL protein, may prove to be an example for other unstable loci that are characterized by recent segmental duplications. As some of these, like the *1q21.1* locus, are associated with disease, it will be intriguing to see if gene conversion also affects genetic configurations of such loci.

Ever since the availability of genomes from Neanderthals, Denisovans, and ancient humans, the question was raised which modern human-specific coding variants may hold clues to how our species evolved over the last ∼600,000 years. Here, we discovered such variants in the *NOTCH2NL* genes, a gene family that emerged in humans about 4 Ma. The role of *NOTCH2NL* genes in human brain development and their involvement in *1q21.1* CNVs associated with a wide variety of neurological disorders emphasizes the importance of the discoveries we describe here: Even if the driving forces of the observed evolutionary changes lie outside the brain, the recent and ongoing structural evolution of human *NOTCH2NL* genes suggests that the tightly coordinated process of human cortical neurogenesis is still subject to fine-tuning.

## Materials and Methods

### *NOTCH2NL* Copy Number Analysis from Whole-Genome Sequencing Data

Fastq files were imported from the EBI SRA to the Galaxy EU or US server (Afgan et al. 2018). For Simons diversity data, only the R1 data were used. Reads were trimmed using Trimmomatic (Galaxy v0.36.5) by the following settings: SLIDINGWINDOW: 4, 20 and MINLEN: 30. The remaining reads were mapped to the *NOTCH2NL*-masked hg38 reference genome using Bowtie2 (Galaxy v2.3.4.2), using single-end, very sensitive end-to-end settings. Sequence read depth per genome was ∼15–30×. The BAM output files were sliced using samtools slice (Galaxy v2.0.1) with the coordinates chr1:118911553–121069626. Bedtools coverage (Galaxy v2.27.0.2) was applied to each sliced BAM file, reporting coverage for each position. The *NOTCH2*-single-copy region used is located at chr1:119908310–119989035, the *NOTCH2 + NOTCH2NL* multicopy region used is located at chr1:119990490–120087745. Each region was filtered for repeats using RepeatMasker, and only the nonrepeat intervals were used in coverage analysis. Mean coverage across both regions was calculated by averaging coverage per position. The mean coverage of the *NOTCH2 + NOTCH2NL*-multicopy region was divided by the mean coverage of the *NOTCH*2-single-copy region to infer *NOTCH2NL* copy-number pet data set. BAM file data were visualized in the UCSC genome browser (Kent et al. 2002). For ancient DNA data sets which consisted of multiple libraries, each library was mapped separately and then merged. The Denisova3 run ERR141700 was omitted due to high sequence duplication. The following WGS data sets were used:

**Table:** 

**Modern human**	
PRJEB9586 (Mallick et al. 2016)	Simons diversity genomes
NA (Van Hout et al. 2019)	UK Biobank exomes
**Ancient human**	
PRJEB6622 (Fu et al. 2014)	Ust’-Ishim
PRJEB6272 (Lazaridis et al. 2014)	Loschbour, StuttgartLBK, Motala3, Motala12
PRJNA240906 (Gamba et al. 2014)	NE1, BR2, IR1, KO1, NE6, NE7, CO1, NE5, BR1
PRJEB4604 (Schiffels et al. 2016)	12880A, 12881A, 12883A, 12884A, 15594A-sc-20
PRJEB21878 (Skoglund et al. 2017)	I9028, I9133, I9134
PRJEB11004 (Martiniano et al. 2016)	3DRIF-16, 3DRIF-26, 6DRIF-18, 6DRIF-21, 6DRIF-22, 6DRIF-23, 6DRIF-3, M1489, NO3423
PRJEB24629 (de la Fuente et al. 2018)	IPK12, IPY10
PRJEB27628 (Krzewińska et al. 2018)	chy002, kzb002, kzb005, kzb006, kzb007, kzb008, mur003, mur004, scy009, scy301, scy303
PRJEB13123 (Fu et al. 2016)	Karelia
PRJEB11364 (Jones et al. 2015)	Bichon, Kotias, Satsurblia
PRJEB21940 (Günther et al. 2018)	Sf12, H22, Sf913, Stg001
PRJEB9783 (Günther et al. 2015)	atp002, atp12-1240
PRJNA218466 (Raghavan et al. 2014)	Mal’Ta
PRJEB21037 (Saag et al. 2017)	Kunila1, Ardu2
PRJEB18067 (Jones et al. 2017)	Latvia_HG1, Latvia_HG2, Latvia_HG3, Latvia_MN2
PRJEB11995 (Cassidy et al. 2016)	BA64, RM127, RSK1, RSK2
PRJEB29663 (Wright et al. 2018)	MH8
PRJEB31045 (Sánchez-Quinto et al. 2019)	ans017, prs016, prs002, prs009
PRJNA338374 (Bhattacharya et al. 2018)	Atacama
PRJEB23467 (Valdiosera et al. 2018)	atp002, atp016
PRJEB7618 (Seguin-Orlando et al. 2014)	Kostenki 14
PRJNA284124 (Rasmussen et al. 2015)	Kennewick
PRJNA46213 (Rasmussen et al. 2010)	Saqqaq
PRJNA229448 (Rasmussen et al. 2014)	Anzick-1
PRJEB6943	Cr10-sc, PA38-sc, PA30-sc
PRJEB2830 (Keller et al. 2012)	Ötzi
PRJNA230689 (Olalde et al. 2014)	La Brana
PRJEB6090 (Skoglund et al. 2014)	Gökhem2, Ajvide58
**Neanderthal**	
PRJEB1265 (Slon et al. 2017)	Altai
PRJEB21157 (Prüfer et al. 2017)	Vindija
PRJEB21195 (Prüfer et al. 2017)	Mezmaiskaya1
NA (Mafessoni et al. 2020)	Chagyrskaya
PRJEB21870 (Hajdinjak et al. 2018)	Goyet Q56-1
PRJEB21875 (Hajdinjak et al. 2018)	Les Cottes Z4-1514
PRJEB21881 (Hajdinjak et al. 2018)	Mezmaiskaya2
PRJEB21882 (Hajdinjak et al. 2018)	Vindija 87
PRJEB21883 (Hajdinjak et al. 2018)	Spy 94a
PRJEB2065 (Green et al. 2010)	Vi33.16, Vi33.25, Vi33.26
**Denisova**	
PRJEB3092 (Meyer et al. 2012)	Denisova3	
PRJEB20653 (Slon et al. 2017)	Denisova2	
**Neanderthal/Denisova hybrid**
PRJEB24663 (Slon et al. 2018)	Denisova11

For comparisons of the SUN analysis with previously assembled *NOTCH2NL* configurations (Fiddes et al. 2018), the following samples and data sets were used (Steinberg et al. 2014; Zook et al. 2016; Eberle et al. 2017; Regier et al. 2018; Audano et al. 2019; Marks et al. 2019):

NA24143: 10× genomics (GIAB), WGS (PRJNA200694), WXS (PRJNA200694)

NA24149: 10× genomics (GIAB), WGS (PRJNA200694), WXS (PRJNA200694)

NA24385: 10× genomics (GIAB), WGS (PRJNA200694, PRJNA428496), WXS (PRJNA200694)

NA19240: WGS (PRJNA288807, PRJNA428496, PRJEB4252)

NA12891: WGS and 10× WGS (PRJEB3381, PRJNA428496, PRJNA393319)

CHM1: WGS (PRJNA246220, PRJNA176729)

### Separation of *NOTCH2NL* Copy Number per Allele Using SUNs

Based on the hg38 reference genome, single-nucleotide variants and indels were identified, via DNA sequence alignment of the *NOTCH2NLA*, *-B*, *-C*, or *-R* loci to the *NOTCH2* locus. Only SUNs within the region chr1:119990474–12008798 were considered, as this is the maximal duplicon size present in each of the *NOTCH2NL* loci based on the segmental duplication track in the UCSC genome browser hg38. The position of each of these SUNs per locus was stored in BED format. These were used to generate .vcf format data per BAM file reporting the total read depth and variant (SUN) depth for these positions. This was done using samtools (v1.7) mpileup:

samtools mpileup -uvf hg38.fasta -t DP -t AD -l variant_positions.bed -Q 13 -q 0 -b datasets.txt > output.vcf

The relevant information to calculate SUN frequency per allele was extracted using bcftools (v1.7) query:

bcftools query -f ‘%CHROM\t%POS\t%REF\t%ALT{0} [\t%DP\t%AD{0}\t%AD{1}]\n’ -H mpileup_output.vcf > mpileup_output_variants.vcf

The frequency per variant was calculated using these output files by dividing allele depth for each SUN (AD) by total depth (DP). For each locus, only SUNs with >0.67 frequency in the population were used for analysis to account for ambiguous or population-specific sites that may skew allele distribution calculation, such as known common SNPs. The frequency of the selected SUNs was averaged per locus and multiplied by the total number of alleles calculated previously based on sequence read coverage, to transform allele frequencies into allele counts. Since there are many SUNs for *NOTCH2*, *NOTCH2NLR*, and *NOTCH2NLC*, they provide an accurate estimation for the allele count of these loci. For *NOTCH2NLA* and *NOTCH2NLB*, only a few SUNs are present and gene conversion phenomena happen frequently, which makes this procedure challenging. Therefore, to analyze these loci, we first subtracted the *NOTCH2*, *NOTCH2NLR*, and *NOTCH2NLC* allele counts from the total allele count. The remaining alleles must be derived from *NOTCH2NLA* and *NOTCH2NLB*, and so, the remaining alleles were counted using the ratio of the average SUN frequency for *NOTCH2NLA* and *NOTCH2NLB*. These data were plotted in donut-charts using LibreOffice v6.1.0.3. For graphs showing the per-SUN allele count across the *NOTCH2NL* loci, the *NOTCH2NLB* SUN count was inferred from the *NOTCH2NLA* SUN count in the 5′ region of the locus, where no *NOTCH2NLB* SUNs are present. For example in modern humans there are four *NOTCH2NLA*+*NOTCH2NLB* loci, then the Exon1^B-(High)^ allele count was calculated according to this: Exon1^B-(High)^ allele count = 4 − Exon1^A-(Low)^ count. Correction for NOTCH2 > NOTCH2NLR gene conversion was done for genomes that showed three alleles NOTCH2. These showed a concordant decrease of one allele NOTCH2NLR based on both the coverage analysis and SUN analysis. This difference was corrected for, in example, three alleles NOTCH2 and one allele NOTCH2NLR in one individual were corrected to two alleles NOTCH2 and two alleles NOTCH2NLR. For separation of the Simons diversity genomes data per population, the sample metadata supplied with the data were used.

### Allele Frequencies in UK Biobank Exome Data

Reads mapping on *NOTCH* or *NOTCH2NL* genes were extracted from UK Biobank CRAM exome files (>20× coverage) mapped on hg38. As in these data sets, the reads are mapped to *NOTCH* and all *NOTCH2NL* loci in hg38, the analysis was adjusted from the original analysis that used the masked hg38. For the Exon1^A-(Low)^ variant (AT**G**>AT**A**), the following positions were analyzed:

**Table:** 

Position	Locus	Orientation	Reference sequence hg38
chr1:120069403–120069404	NOTCH2	−	AT**G**
chr1:120724179–120724180	NOTCH2NLR	+	AT**G**
chr1:146228778–146228779	NOTCH2NLA	−	AT**A**
chr1:148679531–148679532	NOTCH2NLB	−	AT**G**
chr1:149390853–149390854	NOTCH2NLC	+	AT**G**

Similarly, the Exon^2B-(Splice-mut)^ variant information was derived from the following positions:

**Table:** 

Position	Locus	Orientation	Reference sequence hg38
chr1:120029988–120029989	NOTCH2	−	T
chr1:120763625–120763626	NOTCH2NLR	+	A
chr1:146189382–146189383	NOTCH2NLA	−	T
chr1:148640098–148640099	NOTCH2NLB	−	**C**
chr1:149430931–149430932	NOTCH2NLC	+	A

Nea1^N232S^ variant (AAT > AGT) information was derived from the following positions:

**Table:** 

Position	Locus	Orientation	Reference sequence hg38
chr1:119997052–119997053	NOTCH2	−	T
chr1:120793439–120793440	NOTCH2NLR	+	A
chr1:146156535–146156536	NOTCH2NLA	−	T
chr1:148607465–148607466	NOTCH2NLB	−	T
chr1:149463769–149463770	NOTCH2NLC	+	A

Read depth and allele depth analysis using samtools and bcftools was then done for each locus with the following parameters:

samtools mpileup -uvf hg38.fasta -t DP -t AD -l variant_positions.bed -Q 13 -q 0 -b datasets.txt > output.vcf

bcftools query -f ‘%CHROM\t%POS\t%REF\t%ALT{0} [\t%DP\t%AD{0}\t%AD{1}]\n’ -H output.vcf > query_output.vcf

The setting -q (mapping quality) was set to 0, to include multimapping reads that cannot be confidently assigned to a specific *NOTCH2NL* locus but still contain information regarding variant frequencies. Since the Exon1^A-(Low)^ variant is annotated in the hg38 genome in *NOTCH2NLA*, reads containing this variant will map there with a better alignment score. As such, the Exon1^A-(Low)^ frequency was calculated by read depth at the *NOTCH2NLA* position divided by the sum of read depths at the *NOTCH2* + all *NOTCH2NL* loci. The Exon^2B-(Splice-mut)^ frequency was calculated by read depth at the specific *NOTCH2NLB* position, where this variant is annotated in hg38, divided by the total read depth at the paralogous positions.

### Cell Culture

HEK293 cells (ATCC CRL-1573) were cultured in Dulbecco's modified eagle medium (DMEM) + GlutaMax, high glucose (Thermofisher 61965026), supplemented with 10% heat-inactivated fetal bovine serum (HIFBS) (Thermofisher 10500064) and 100 µg/ml Pen/Strep (Thermofisher 15140122). U2OS and U2OS-JAG2 cells (gifts of Arjan Groot and Marc Vooijs, MAASTRO Lab, Maastricht University) were cultured in DMEM + GlutaMax, high glucose, supplemented with 10% HIFBS and 100 µg/ml Pen/Strep. U2OS-JAG2 cells were additionally supplemented with 2 μg/ml puromycin (Sigma P8833). For routine passaging, medium was removed and cells washed once with phosphate-buffered saline (PBS) (Thermofisher 10010056). A sterile filtered 0.25% Trypsin (Thermofisher 15090046) + 0.5 mM disodium-ethylenediaminetetraacetic acid (EDTA) (Sigma E5134) solution in PBS was added, and incubated at 37 °C for 2 min. One-eighth of the cell suspension was transferred to a new culture vessel of the same size.

### Transfection for NOTCH2NL Variant Protein Analysis

HEK293 cells were seeded 24 h before transfection in a six-well plate. One hour before transfection, medium was replaced with 1,800 μl DMEM + GlutaMAX, high glucose and 10% HIFBS. The transfection mix per well was as follows: 500 ng of pCAGN1-NOTCH2NL or pCAGN1-EV, and 500 ng of pCAGEN-GFP were mixed in a total volume of 100 μl 0.25 M CaCl_2_, after followed by addition of 100 μl 2× HEPES-buffered saline (50 m HEPES, 1.5 mM Na_2_HPO_4_, 140 mM NaCl, pH 7.05). The 200 μl solutions were mixed by pipetting up and down five times, and the complete mix was added to one well of a six-well plate. Six hours after adding transfection mixes, medium was replaced.

### Protein Isolation

Cells were isolated for protein extraction 24–30 h after transfection. Cells were washed twice in ice-cold PBS, then detached using a cell scraper (VWR 734-1527) and transferred to 1.5-ml microcentrifuge tubes. Cell suspensions were centrifuged at 4 °C for 5 min at 1,000 rcf to pellet cells. The supernatant was removed and the cells resuspended in 10× the pellet volume (100–150 μl) of immunoprecipitation lysis buffer (50 mM Tris–HCl pH8.0, 150 mM NaCl, 5 mM MgCl_2_, 0.5 mM EDTA, 0.2% NP40 substitute, and 5% glycerol), supplemented with 1× protease inhibitor cocktail (Sigma 5892791001). After incubating for 1 h at 4 °C, cell suspensions were transferred through a 273/4 gauge needle ten times and centrifuged at 20,817 rcf for 10 min at 4 °C to pellet cell debris. The supernatant was transferred to a new 1.5-ml microcentrifuge tube and stored at −80 °C.

### Protein Gel Electrophoresis and Western Blot

Twenty microliters of protein extract was mixed with 20 μl of 2× laemmli sample buffer (Biorad 1610737) + 50 mM DTT (Sigma D0632). Samples were heated for 5 min at 95 °C and briefly centrifuged. Twenty microliters per sample was loaded on a 1.5-mm poly-acrylamide gel, consisting of two parts. The running gel (12% acrylamide/Bis, 375 mM Tris–HCl pH 8.8, 0.1% ammonium persulfate [APS], 0.1% sodium dodecyl sulfate [SDS], and 0.04% tetramethylethylenediamine [TEMED]) and the stacking gel (5% acrylamide/Bis, 0.125 mM Tris–HCl pH 6.8, 0.1% APS, 0.1% SDS, and 0.1% TEMED). Twenty microliters of sample was loaded per well and 5 μl of marker (Thermofisher #26619) was used for reference. Electrophoresis was done in 25 mM Tris + 192 mM glycine buffer (Biorad 1610771) and 0.1% SDS. Protein was transferred to nitrocellulose membrane (Sigma 10600004), at 100 V for 2 h in Towbin buffer (25 mM Tris, 192 mM glycine, and 20% methanol). Blots were rinsed three times with demi-water, and transfer was checked by ponceau S staining. Blots were rinsed once in Tris buffered saline (20 mM Tris, pH 7.5, 150 mM NaCl) + 0.1% Tween (TBS-T), followed by incubation in blocking buffer (TBS-T + 5% w/v skim milk powder) for 90 min at room temperature on a shaking platform. Primary antibodies were incubated overnight at 4 °C in TBS-T in 50-ml tubes on a tube roller. Antibodies used were rabbit anti-HA tag (1:6,000, Abcam ab9110) or rabbit anti-GFP (1:4,000, Abcam ab290). Blots were rinsed once in TBS-T and washed in TBS-T three times 15 min on a shaking platform. Secondary antibody goat anti-rabbit-HRP in TBS-T (1:20,000, Thermofisher 656120) was incubated for 60 min at room temperature. Blots were rinsed once in TBS-T and washed three times 15 min in TBS-T on a shaking platform. The SuperSignal Westdura substrate (Thermofisher 34075) was used for chemiluminescent detection, imaged with a ChemiDoc MP imaging system (Biorad 1708280). Signals were quantified using Fiji ImageJ using the NOTCH2NL/GFP ratio.

### Coculture NOTCH Reporter Assay

To monitor modulation of NOTCH2 activity by NOTCH2NL, a reporter assay was used. The pGL3-UAS luciferase reporter can be activated by S3-cleaved NOTCH2-Gal4-N1TAD receptor intracellular domain (Gal4 domain fused to NOTCH1-transactivation domain) (gifts of Arjan Groot and Marc Vooijs, MAASTRO Lab, Maastricht University). To achieve high levels of receptor activation, the cells transfected with pcDNA5-NOTCH2-Gal4-N1TAD are cocultured with JAG2 expressing cells. Coculture with regular U2OS cells was done as a control. pCAGN1-EV or pCAGN1-NOTCH2NL (derived from Addgene 51142) were cotransfected to measure effects of NOTCH2NL on reporter activity. pRL-CMV (Promega E2261) was used for normalization.

For transfection, U2OS cells were seeded in six-well plates at a density of 400,000 cells per well. For coculture assay, U2OS cells or U2OS-JAG2 cells were seeded in 12-well plates at a density of 110,000 cells per well. Twenty-four hours later, U2OS cells in six-well plates were transfected. The transfection complex per well was made by adding 2,500 ng plasmid DNA mix, as described in the table below, in 100 μl OptiMEM (Thermofisher 31985047). In a different tube, 8.33 μl PEI (1 mg/ml, Polysciences 23966) was added to 100 μl OptiMEM. One hundred microliters of each mix was combined, incubated 20 min at room temperature, and added to the well containing 2 ml of complete medium. Reactions were scaled accordingly to facilitate large-scale transfections. Six hours after transfection, the transfected cells were replated onto the 12-wells plate for coculture with U2OS or U2OS-JAG2 cells. Per well, medium was removed and cells were washed once with 1 ml PBS. Trypsin-EDTA (0.5 ml) in PBS was added plates were incubated 90 s at 37 °C. Two millilietrs of complete medium was added, and cell aggregates were broken up by pipetting up and down three times. Cell suspension was transferred to 15-ml conical tubes already containing 4.5 ml of complete medium. From the 12-well plates, the medium was removed and replaced by 1 ml of cell suspension. To control wells, 1 μl of 200 μM DBZ was added. Twenty-four hours after replating, the cells were isolated for luciferase assays using Dual-Luciferase Reporter Assay System (Promega E1980). Medium was removed and each well washed once with 0.5 ml PBS. A total of 150 μl of 1× passive lysis buffer (Promega E1941) was added per well and incubated 15 min on a rotating platform. Plates were wrapped in parafilm and stored at −80 °C. For analysis, 20 μl sample was pipetted to a 96-well optiplate (PerkinElmer 6005290). Samples were measured on a GloMax Navigator device (Promega GM2010), with the following settings: Injector 1, LARII buffer (volume 50 μl, speed 200 μl/s). Wait 2 s. Measure luminescence Luciferase (integration 10 s, readings 1, interval 0.3 s). Injector 2, Stop & Glo buffer (volume 50 μl, speed 200 μl/s). Wait 2 s. Measure luminescence Renilla (integration 10 s, readings 1, interval 0.3 s). For comparison of human, Neanderthal and Denisovan NOTCH2NL variants, the 48 ng pCAGN1-NOTCH2NL condition was used.

**Table:** 

	6	16	48	140	420
**pGL3-UAS**	1,050	1,050	1,050	1,050	1,050
**pRL-CMV**	70	70	70	70	70
**pCAGEN-GFP**	35	35	35	35	35
**pcDNA5-NOTCH2-Gal4-N1TAD**	21	21	21	21	21
**pCAGN1-EV/-NOTCH2NL**	5/6	14/16	41/48	120/140	361/420
**pBluescript (EV/NOTCH2NL)**	1,315/1,314	1,306/1,304	1,279/1,272	1,200/1,180	959/900

Amount of plasmid DNA (ng) transfected per condition. pCAGN1-EV/pCAGN1-NOTCH2NL denotes amount of plasmid used per condition accounting for molarity. pBluescript amount was adjusted accordingly as well.

### In Silico Analysis of Archaic Coding Variants

For multiple sequence alignment of NOTCH1, -2, and -3 EGF-L domains 6, the relevant sequences were acquired from UniProt and compared using the alignment tool of UniProt. The EGF-L repeat domain consensus sequence was retrieved from Prosite: PDOC00021, EGF_3 PS50026. For MutPred2 and IUPred2A analysis, the archaic amino acid variants were introduced in the NOTCH2NLB protein sequence retrieved from UniProt (P0DPK3). MutPred2 was run with a *P* value threshold of 0.05. IUPred2A was used with the following settings: Long disorder, Context-dependent predictions: ANCHOR2.

**Table:** 

Plasmids
pCAGEN-GFP (Addgene #11150)
pCAGN1- hCas9 (Addgene #51142)
pCAGN1- EV
pCAGN1-NOTCH2NL
pCAGN1-NOTCH2NL-T197I
pCAGN1-NOTCH2NL-M40I, T197I
pCAGN1-NOTCH2NL-N232S, T197I
pCAGN1-NOTCH2NL-E258A, T197I
pCAGN1-NOTCH2NL-M1I
pCAGN1-NOTCH2NL-M1I, T197I
pCAGN1-NOTCH2NL-M1I, M40I
pCAGN1-NOTCH2NL-M1I, N232S
pCAGN1-NOTCH2NL-M1I, E258A
pCAGN1-NOTCH2NL-HA
pCAGN1-NOTCH2NL-HA-T197I
pCAGN1-NOTCH2NL-HA-M40I, T197I
pCAGN1-NOTCH2NL-HA-N232S, T197I
pCAGN1-NOTCH2NL-HA-E258A, T197I
pCAGN1-NOTCH2NL-HA-M1I
pCAGN1-NOTCH2NL-HA-M1I, T197I
pCAGN1-NOTCH2NL-HA-M1I, M40I
pCAGN1-NOTCH2NL-HA-M1I, N232S
pCAGN1-NOTCH2NL-HA-M1I, E258A
pCAGN1-NOTCH2NL-HA-5′ M1
pCAGN1-NOTCH2NL-HA-5′ M1 + kozak
pCAGN1-NOTCH2NL-HA-5′ M40
pCAGN1-NOTCH2NL-HA-5′ M40 + kozak
pCAGN1-NOTCH2NL-HA-5′ M84
pCAGN1-NOTCH2NL-HA-5′ M84 + kozak
pCAGN1-NOTCH2NL-HA-5′ M1I-I1
pCAGN1-NOTCH2NL-HA-5′ P2
pCAGN1-NOTCH2NL-HA-5′ L12
pCAGN1-NOTCH2NL-HA-5′ P22
pCAGN1-NOTCH2NL-HA-5′ C28
pCAGN1-NOTCH2NL-HA-M1I-ΔI1
pCAGN1-NOTCH2NL-HA-M1I-ΔL4
pCAGN1-NOTCH2NL-HA, 5′ M1, CTG^(1-5)^ > CTA^(1-5)^
pCAGN1-NOTCH2NL-M1I-HA, 5′ I1, CTG^(1-5)^ > CTA^(1-5)^
pCAGN1-NOTCH2NL-M1I-HA, 5′ I1, Δata-CTG^(1-5)^ > CTA^(1-5)^
pcDNA5-NOTCH2-GAL4-TAD-N1
pRL-CMV (Promega E2261)

### Statistics

Luciferase reporter assay data were first analyzed using analysis of variance (ANOVA) by the R function “aov().” Significant groups were further tested with Tukey’s test using the R function “TukeyHSD().” Western blot data were analyzed in the same way, except for data presented in figure 3*B* and *C*, which showed unequal variance (Levene test *P* = 0.002) and were analyzed instead using Welch corrected ANOVA using the R functions “oneway()” with parameters “levene=TRUE” and “corrections=TRUE,” followed by Games–Howell test from function “posthocTGH(),” with parameter “method=games-howell” (R package “userfriendlyscience”). Population genetic data from Simons diversity genomes and UK Biobank exomes were first analyzed using Kruskal–Wallis tests by the R function “kruskal.test().”Significant groups were further tested with Dunn’s test, using the “dunn.test()” function (R package “dunn.test”). Distributions in figure 5*C* and supplementary figure S5*A*, Supplementary Material online, were tested using the Kolmogorov–Smirnov test using the “ks.test()” function. Expected distributions were generated using the “rnorm()” function in R. For Simons data, this was simulated by generating mean allele counts according to an AABB × AABB polygenic inheritance pattern for the Exon1A-(Low) and Exon1B-(High) variants: 0 alleles (1/16), 1 allele (4/16), 2 alleles (6/16), 3 alleles (4/16), or 4 alleles (1/16), total *N* = 2,790. Standard deviation was set to 0.34 to introduce sampling variation. Expected distribution in the UK Biobank analysis was done similarly, except using allele frequencies instead, of 0, 0.1, 0.2, 0.3, or 0.4, total *N* = 50,000, with a standard deviation of 0.034, which were adjusted for loss of *NOTCH2NLC* and *NOTCH2NLR* as identified in Simons diversity genomes. Boxplots show median and interquartile range (25th and 75th percentiles), whiskers are defined by 1.5 × interquartile range. Outliers were hidden in violin/box plots from figure 6 and supplementary figures S6–S7, Supplementary Material online, to avoid clutter. All *P* values shown were adjusted for multiple testing using Holm’s method.

### Data Visualization

For donut-charts showing *NOTCH2NL* allele counts, LibreOffice v6.1.0.3 was used. Data involving genomic context were visualized on the UCSC genome browser and exported as.pdf files. Plots showing quantification of sequence read coverage, luciferase assays, Western blots, per-SUN count graphs, variant allele counts, and distributions were generated in RStudio v1.1.463 and R v3.5.3 with the ggplot2 package v3.1.0. Fig. panels were assembled in Adobe Illustrator v23.0.3.

## Data Availability

All genomics data from the Simons Diversity Cohort and Archaic genomes were downloaded from their original depositories. Accession numbers and unique identifiers are provided where necessary. All data from the analyses in this manuscript are included in this published article (and its Supplementary Material online). The raw data from the UK Biobank are not publicly available due to restrictions, but the analyzed data as described in this manuscript are available upon request from the corresponding author. Please note that for UKB analyses we can only share summarized data. Individual-level data may be accessed by submitting an application to UKB.

## Code Availability

All code and software used in this manuscript are described and/or available in the Materials and Methods section.

## Supplementary Material

Supplementary data are available at *Molecular Biology and Evolution* online.

## Supplementary Material

msaa104_Supplementary_Data
