# Supplementary figures and images for: Evolution of Human Brain Size-Associated NOTCH2NL Genes Proceeds toward Reduced Protein Levels
Source: Mol Biol Evol. 2020 Apr 24;37(9):2531–48. doi: 10.1093/molbev/msaa104 (PMC7475042; doi:10.1093/molbev/msaa104)

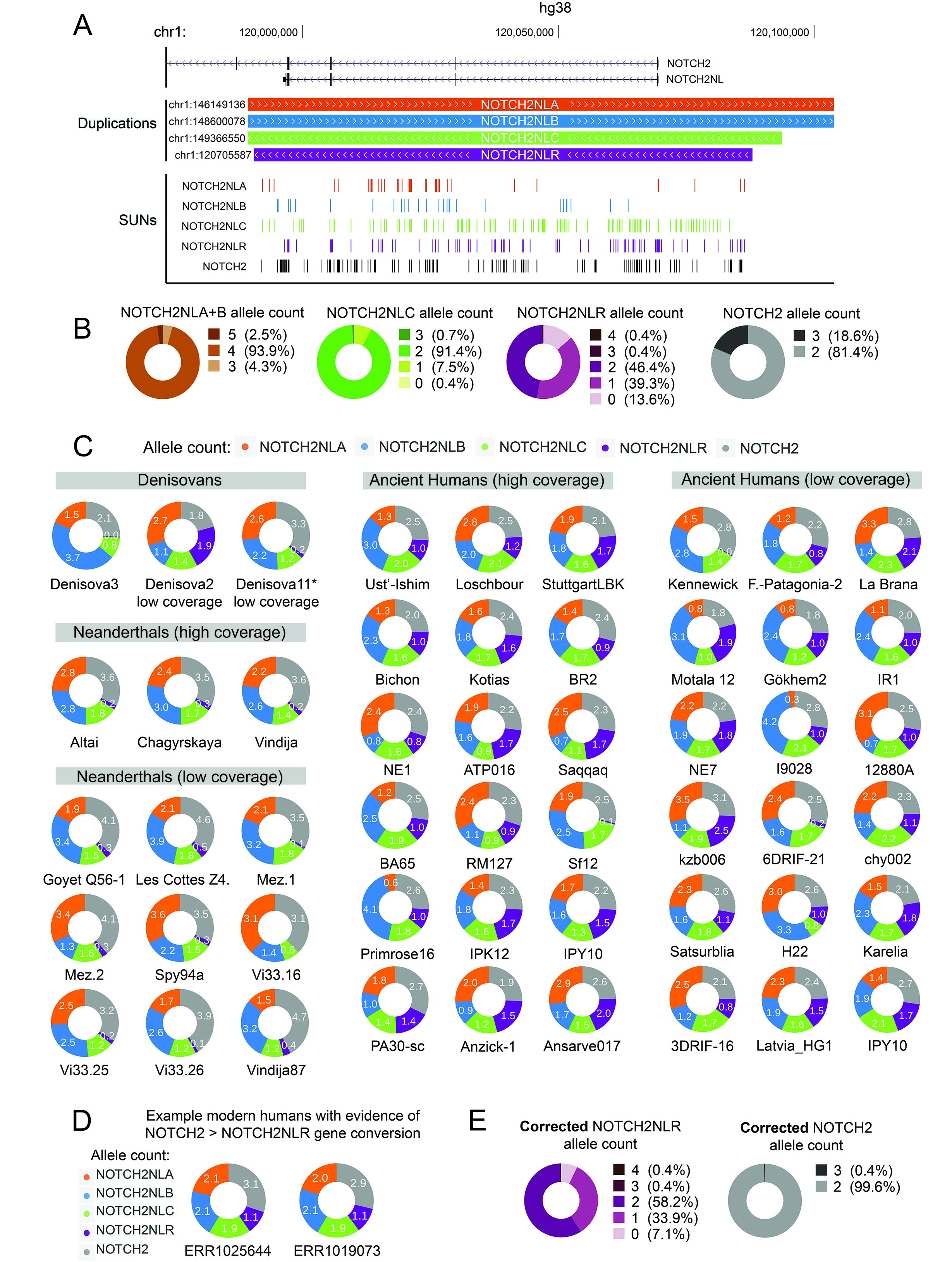

Supplement: msaa104_Supplementary_Data [file msaa104_supplementary_data.zip › msaa104-Suppl_Data/supp_figure1_v08.jpg]

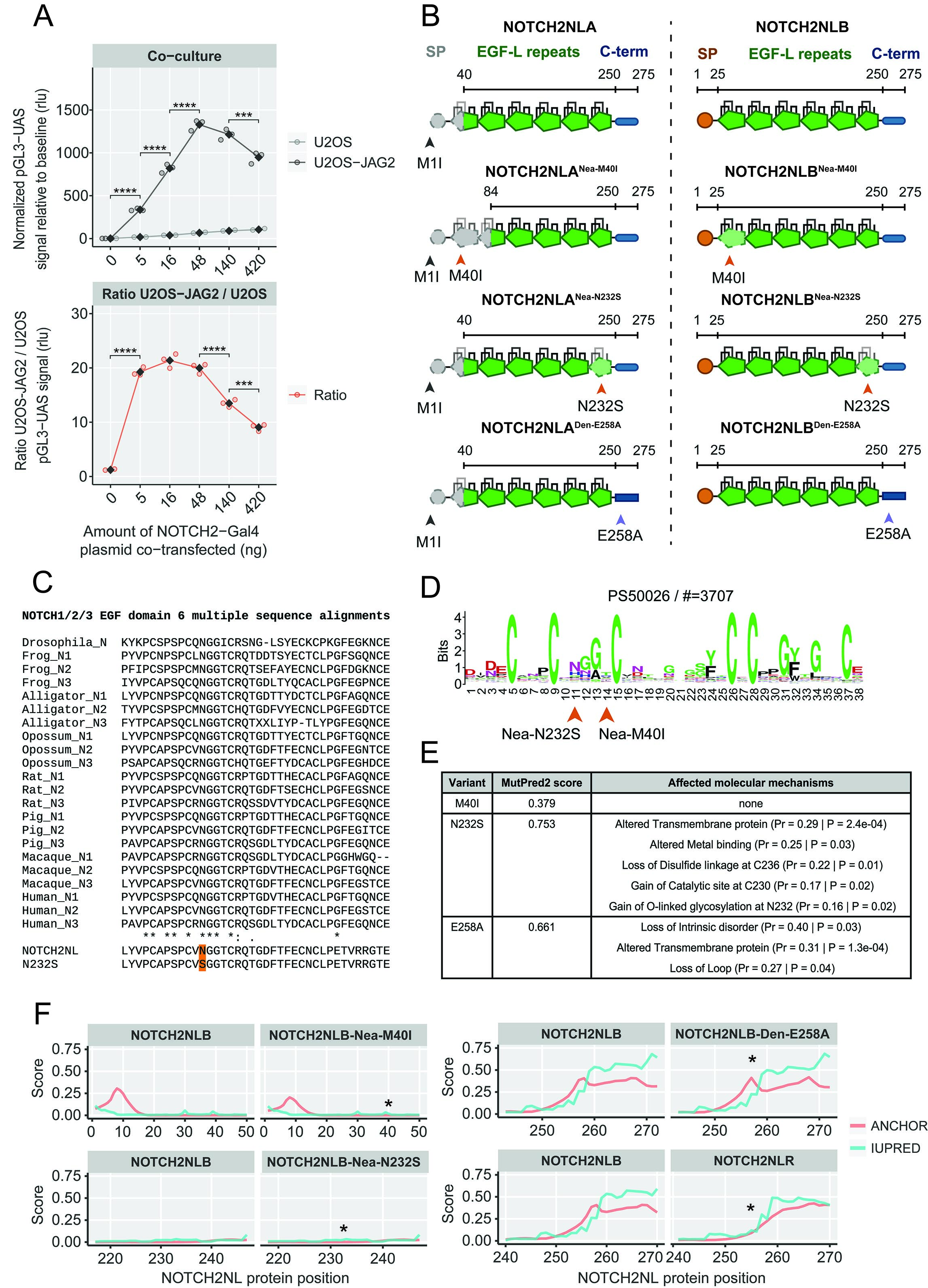

Supplement: msaa104_Supplementary_Data [file msaa104_supplementary_data.zip › msaa104-Suppl_Data/supp_figure2_v06.jpg]

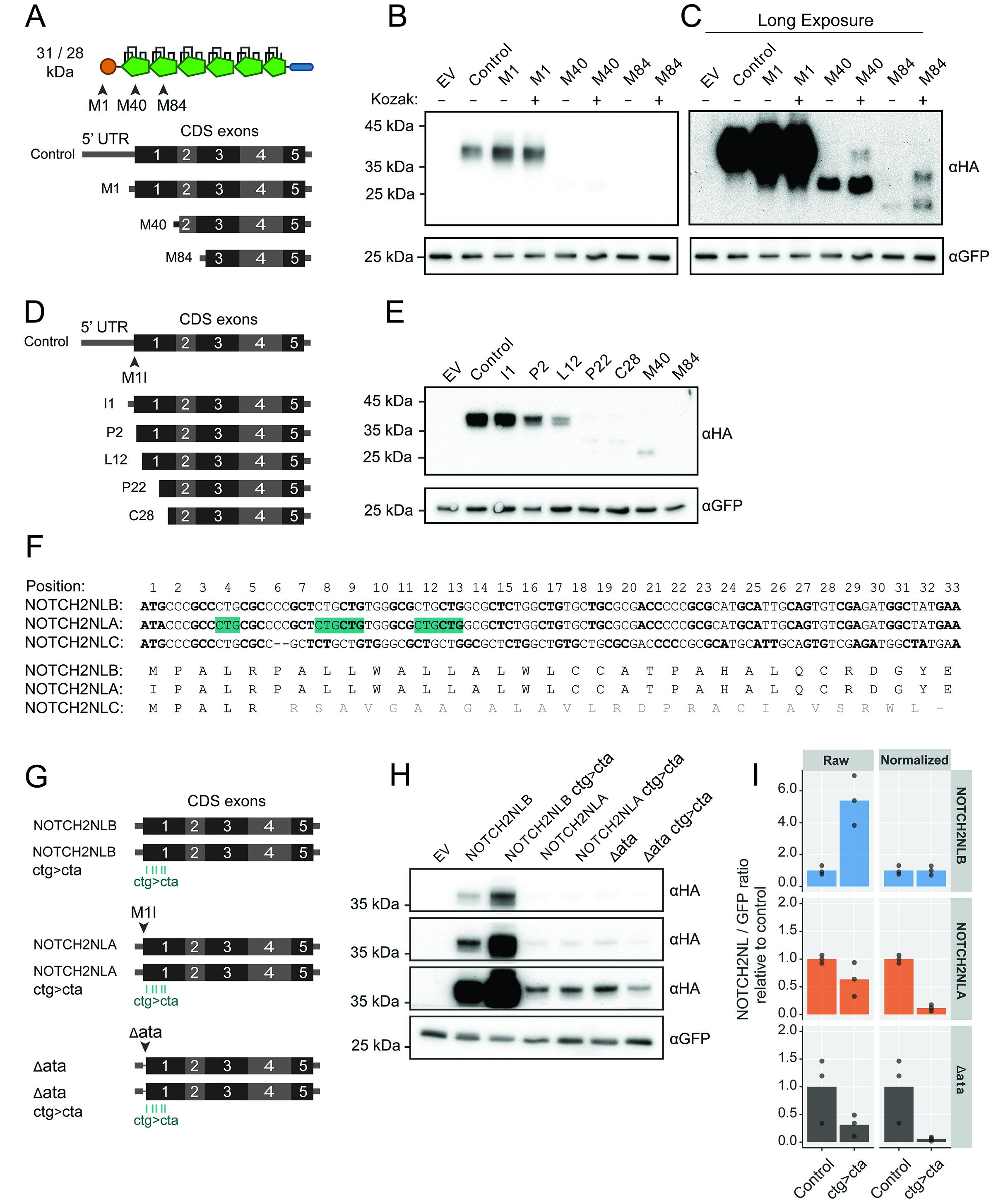

Supplement: msaa104_Supplementary_Data [file msaa104_supplementary_data.zip › msaa104-Suppl_Data/supp_figure3_v06.jpg]

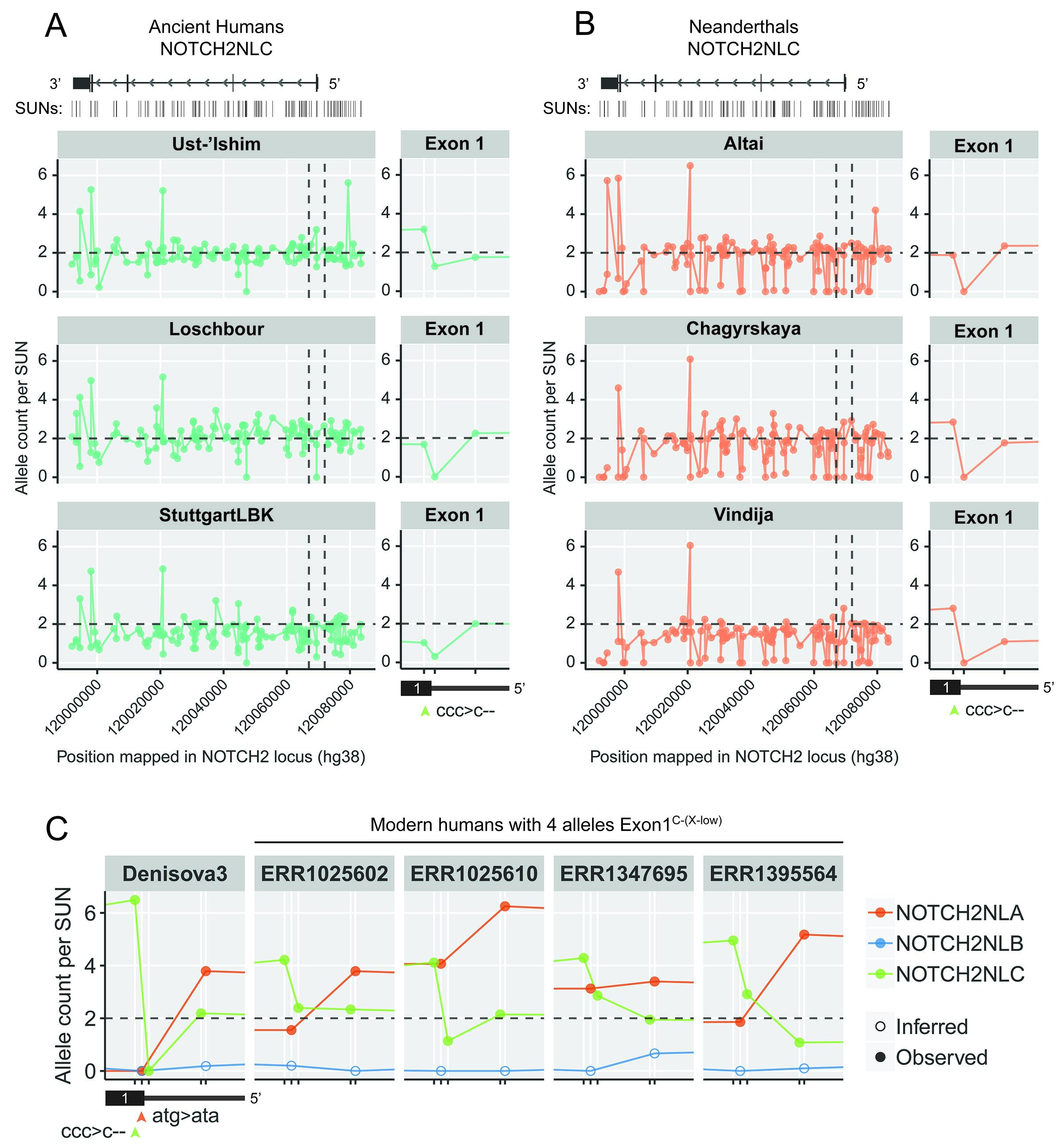

Supplement: msaa104_Supplementary_Data [file msaa104_supplementary_data.zip › msaa104-Suppl_Data/supp_figure4_v04.jpg]

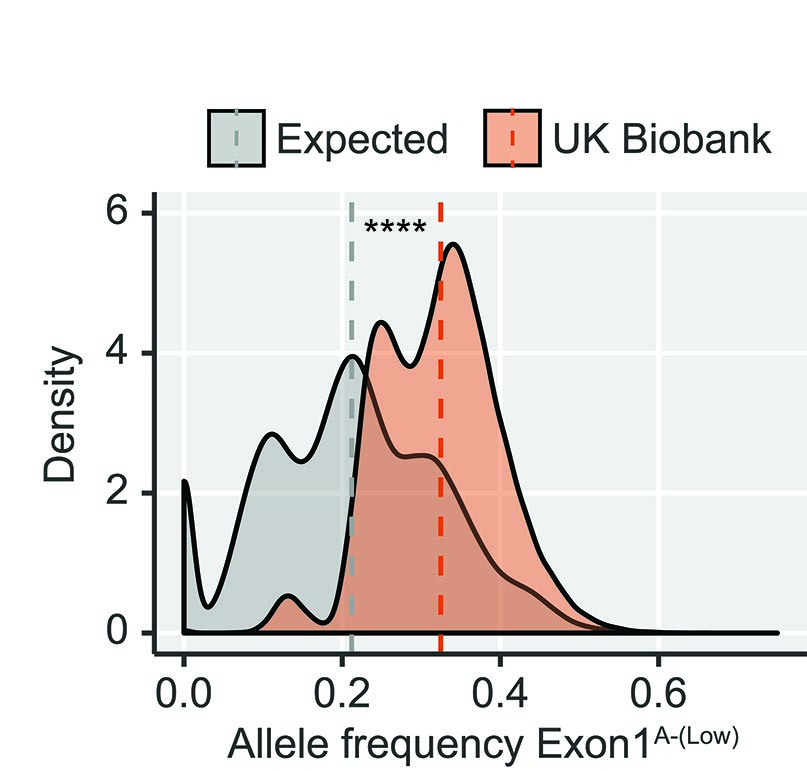

Supplement: msaa104_Supplementary_Data [file msaa104_supplementary_data.zip › msaa104-Suppl_Data/supp_figure5_v05.jpg]

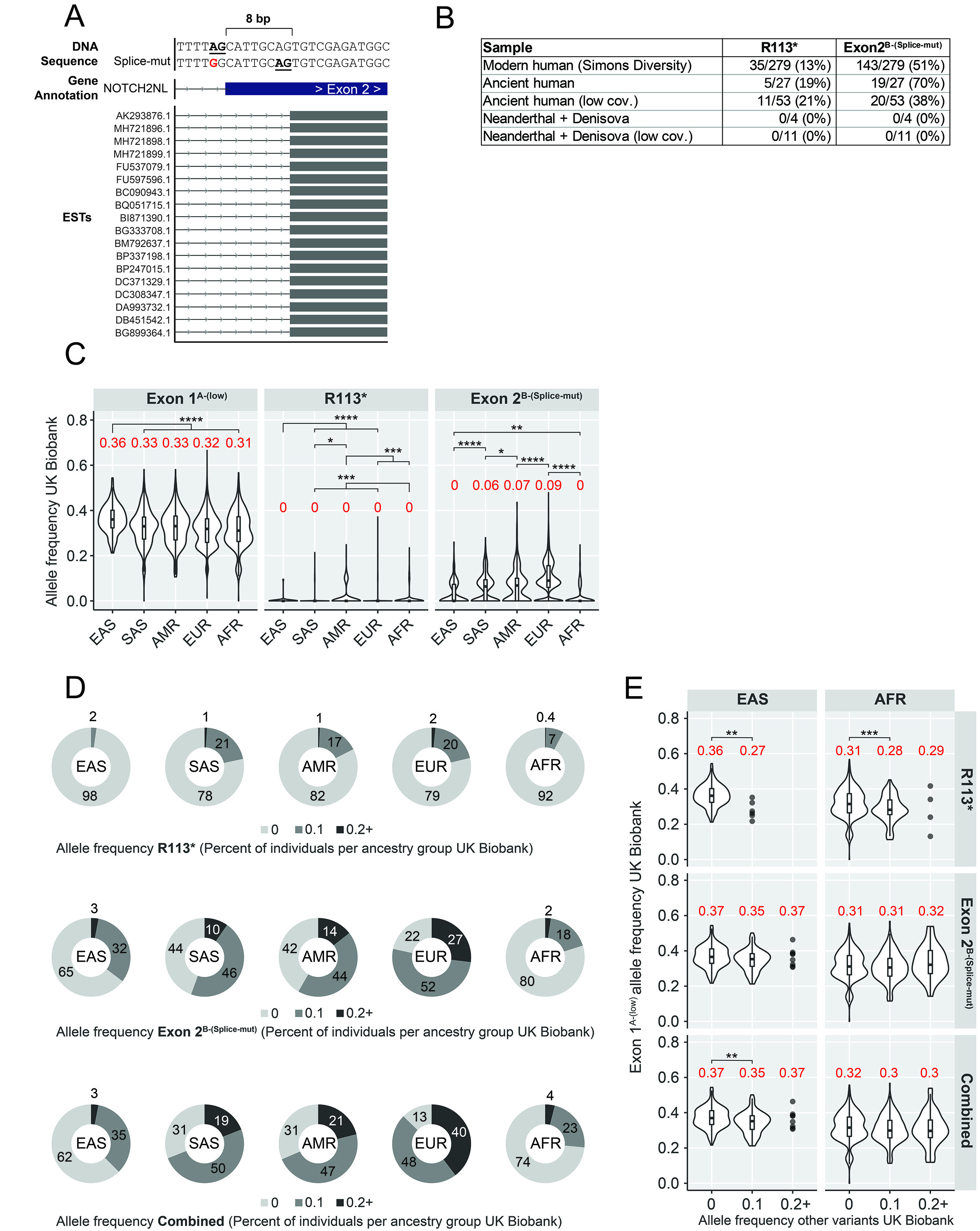

Supplement: msaa104_Supplementary_Data [file msaa104_supplementary_data.zip › msaa104-Suppl_Data/supp_figure6_v10.jpg]

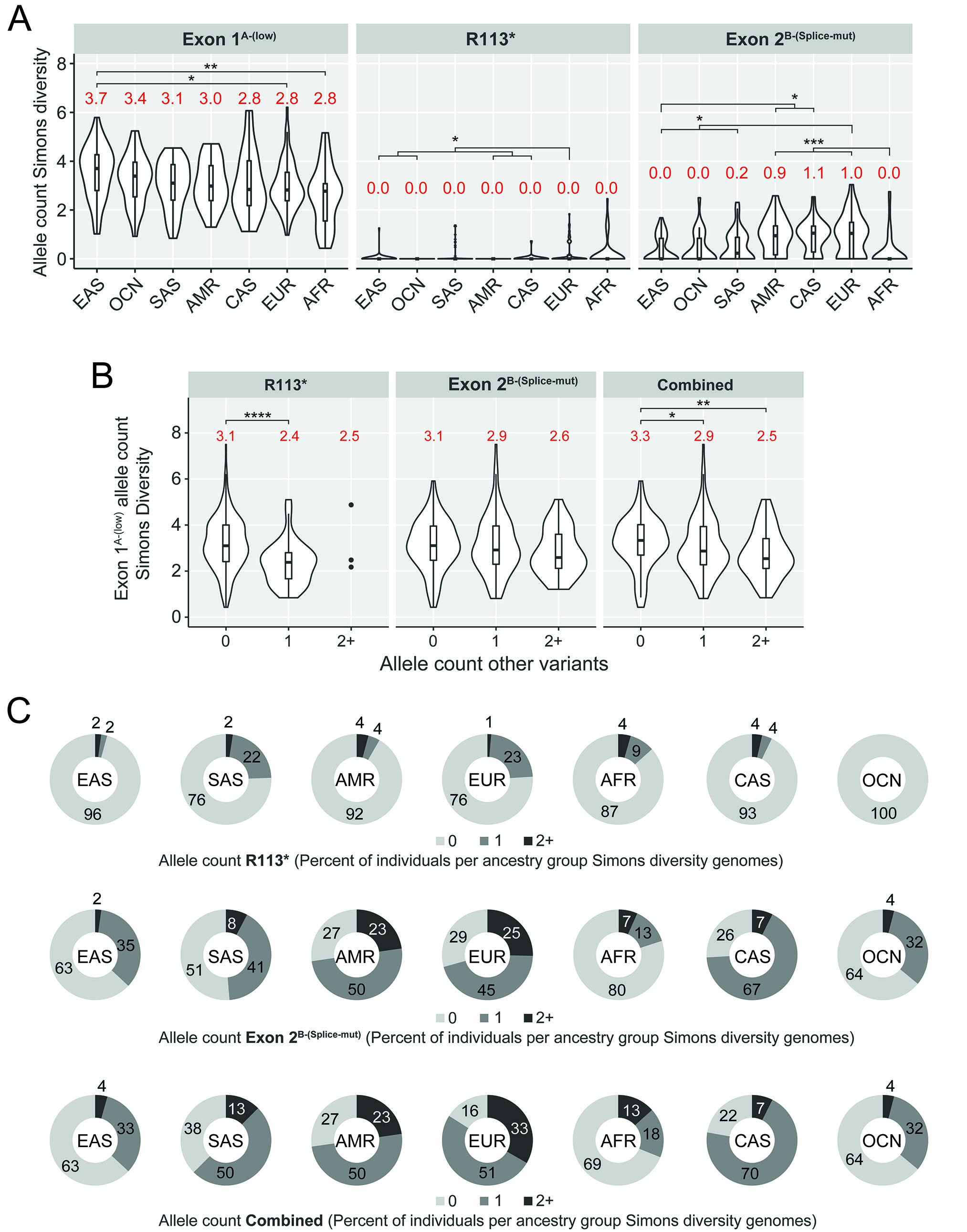

Supplement: msaa104_Supplementary_Data [file msaa104_supplementary_data.zip › msaa104-Suppl_Data/supp_figure7_v03.jpg]
